# Probiotics and muscle health: the impact of *Lactobacillus* on sarcopenia through the gut-muscle axis

**DOI:** 10.3389/fmicb.2025.1559119

**Published:** 2025-03-14

**Authors:** Jingjun Zhu, Fei Peng, Huixin Yang, Jing Luo, Li Zhang, Xiaolong Chen, Huazhi Liao, Hao Lei, Shuai Liu, Tingqian Yang, Guanghua Luo, Guodong Chen, Heng Zhao

**Affiliations:** ^1^Department of Radiology, The First Affiliated Hospital, Hengyang Medical School, University of South China, Hengyang, Hunan, China; ^2^Changde Hospital, Xiangya School of Medicine, Central South University (The First People’s Hospital of Changde City), Changde, China; ^3^Department of Hepatobiliary Pancreatic Surgery, The First Affiliated Hospital, Hengyang Medical School, University of South China, Hengyang, China; ^4^Department of Radiology, The Seventh Affiliated Hospital, Sun Yat-sen University, Shenzhen, Guangdong, China

**Keywords:** sarcopenia, gut-muscle axis, gut microbiota, *Lactobacillus*, muscle

## Abstract

Sarcopenia refers to the decline in skeletal muscle mass and function. Due to its increased mortality rate and severe disability, the clinical importance of sarcopenia is becoming increasingly prominent. Although the exact cause of sarcopenia is not fully understood, the gut microbiota (GM) plays a crucial role in the pathogenesis of sarcopenia, and increasing evidence suggests that gut dysbiosis may be associated with disease development. In the past few decades, the use of probiotics has surged, few studies have explored their impact on sarcopenia prevention and treatment. *Lactobacillus* probiotics are commonly used for gut health and immune support, but their mechanism in sarcopenia via the gut-muscle axis remains uncertain. This review highlights the treatment challenges, GM’s role in sarcopenia, and the potential of *Lactobacillus* as an adjunct therapy. In addition, we also discuss the possible mechanisms by which *Lactobacillus* affect muscle function, such as alleviating inflammatory states, clearing excessive reactive oxygen species (ROS), improving skeletal muscle metabolism, enhancing intestinal barrier function and modulating the gut microbiota and its metabolites. These mechanisms may collectively contribute to the preservation of muscle mass and function, offering a promising avenue for advancing microbial therapies for sarcopenia.

## 1 Introduction

Sarcopenia, as defined by the European Working Group on Sarcopenia in Older People (EWGSOP), refers to a decline in muscle mass and function in terms of strength or performance (Cruz-Jentoft et al., 2019). Due to the aging of the body, muscle mass and strength naturally decrease. In individuals with sarcopenia, this process accelerates significantly, leading to a rapid deterioration in muscle function and eventual development of muscle atrophy. These changes result in a decline in mobility, increasing susceptibility to falls, fractures, disability, and even rising mortality rate (Gomes et al., 2017; Walston, 2012). With the aggravation of population aging and the change of lifestyle, the prevalence of sarcopenia shows a rising trend year by year. Significantly, the prevalence of sarcopenia is approximately 10%–50% in people aged ≥ 60 years, which has become a serious challenge for global health (Witham and Aihie Sayer, 2016). Therefore, the prevention and intervention of sarcopenia are crucial to mitigating its progression and reducing the risk of severe conditions and complications.

It is challenging to ensure timely diagnosis and intervention for sarcopenia. The effectiveness of medications is often limited, and this may even cause side effects. Moreover, the expensive treatment costs combined with individual differences have led to varying outcomes in rehabilitation and nutritional interventions (Cho et al., 2022). Encouragingly, the innovative theory of the gut-muscle axis has become a focal point of research in the scientific community, with its potential to revolutionize our understanding of the interaction between gut health and muscles. According to the “Gut-muscle axis Hypothesis,” muscle function and metabolism largely depend on the quantity and structure of the gut microbiota. It suggests that gut microbes could become potential biological targets for the prevention and therapy of muscle-related disorders such as sarcopenia and muscle atrophy (Ticinesi et al., 2017). Multiple lines of evidence indicate the interaction between gut microbiota and skeletal muscle (Kang et al., 2021; Lahiri et al., 2019; Manickam et al., 2018; Qiu et al., 2021). Hence interventions targeting gut microbiota imbalance, such as dietary changes, supplements, and active compounds, may alleviate sarcopenia. Understanding how the gut microbiota regulates muscle function is especially crucial for enhancing patients’ mobility and strength.

*Lactobacillus*, an important bacterium within the Firmicutes phylum, has attracted significant interest due to its prominent presence in the gut microbiota. Its subgroups have been suggested to influence the progression of sarcopenia, showing protective effects in studies conducted on mice, cells, and humans (Kim et al., 2023; Lee et al., 2023; Ni et al., 2019; Rondanelli et al., 2022). Despite the promising potential of *Lactobacillus*, to the best of our knowledge, a clear and comprehensive review of its specific mechanisms of action in sarcopenia remains absent. Consequently, we elaborate the role of *Lactobacillus* in sarcopenia, attempting to provide new insights into potential mechanism for developing effective therapies for sarcopenia.

## 2 The dilemma of drugs treatment for sarcopenia

Currently, though non-pharmacological treatment such as exercise and nutritional interventions are still the first-line treatment for sarcopenia. Clinicians, pharmaceutist, researchers, etc. have made arduous explorations of drugs for treating sarcopenia. Unfortunately, due to the complex pathogenesis of sarcopenia, there is still no specific drugs approved for treating sarcopenia. Some drugs used in clinical practice to treat other diseases may benefit muscles and expand their use for sarcopenia. Although these drugs have shown good clinical efficacy in treatment, several adverse outcomes that may arise after treating sarcopenia cannot be ignored. For example, Testosterone promotes muscle cell proliferation via the Ras/MEK/ERK pathway and increases muscle mass and strength (De Spiegeleer et al., 2018), but the treatment risks include thrombosis, sleep apnea, and cancer (Morley, 2016). Selective androgen receptor molecules (SARMs) may lead to severe liver and kidney damage, and also result in testicular shrinkage and male infertility (Efimenko et al., 2022). In addition, prolonged administration of myostatin inhibition (MI) may result in raising the risk of cardiovascular disease (Campbell et al., 2017). Growth Hormone (GH) supplementation increases muscle mass but does not enhance strength or function (Papadakis et al., 1996), and is relatively more expensive than other sarcopenia medications (Liu et al., 2007). Overall, there is no safe or effective treatment plan for the medication used to treat sarcopenia. How to innovate and optimize the safety and efficacy of treatment in clinical practice, while ensuring they are affordable for patients with sarcopenia, remains a critical issue that needs to be addressed.

## 3 Probiotics *Lactobacillus* as a key role in the gut-muscle axis

The gut-muscle axis represents a crucial biological mechanism, highlighting the pivotal contribution of gut microbes in maintaining overall lean mass, skeletal muscle mass, and bodily functions. In recent years, considerable valuable results on the gut-muscle axis have been revealed (Liu Y. et al., 2023). First, germ-free mice, which lacked a gut microbiota showed a more pronounced reduction in muscle mass, quality, and neuromuscular performance compared to pathogen-free mice that had a gut microbiota (Lahiri et al., 2019). Second, mice treated with antibiotics experienced increased muscle atrophy, which was associated with an imbalance in gut microbiota and lower levels of ileal fibroblast growth factor 15 (FGF15) were observed, while administering FGF19 effectively reversed muscle atrophy (Manickam et al., 2018; Qiu et al., 2021). Third, sarcopenia patients showed a higher abundance of *Lactobacillus* compared to healthy controls and a decrease of *Eubacterium*, *Lachnospira*, *Fusicatenibacter*, and *Roseburia* genera (Kang et al., 2021). Notably, probiotics are defined as “living microorganisms” by the Food and Agriculture Organization (FAO) and the World Health Organization (WHO), which exert their beneficial effects in the human body by inhibiting the proliferation of pathogenic microbes, generating bioactive compounds, and sustaining a balanced local micro-environment (Hill et al., 2014). *Lactobacillus* is the most researched and commonly utilized probiotic that has been applied in the commercial field for a long time, which can survive in the acidic environment of the gastrointestinal tract (Corcoran et al., 2005). Importantly, the majority of *Lactobacillus*’ subspecies have benefits for gut health, particularly with the potential to help the host restore muscle mass and function.

**FIGURE 1:**
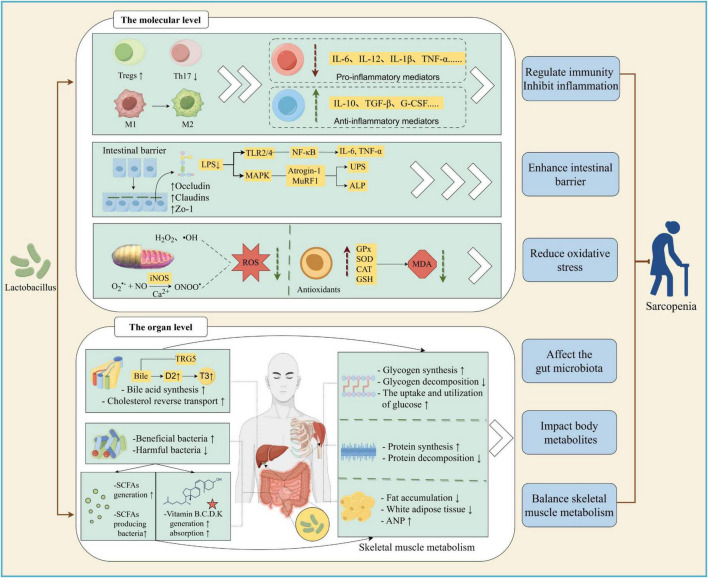
Possible mechanism of *Lactobacillus* alleviating sarcopenia. *Lactobacillus* can alleviate sarcopenia in various ways. (1) At molecular level, lactobacilli alter the phenotype and quantity of immune cells; enhancing gut barrier affects MAPK and NF-KB signaling pathway, thereby reducing the level of inflammatory factors. It can also regulate the level of antioxidant enzymes, thereby reducing reactive oxygen species (ROS) and malondialdehyde (MDA), inhibiting Nitric Oxide (NO) production, alleviating oxidative stress, and ameliorating sarcopenia. (2) At tissue level, *Lactobacillus* can increase skeletal muscle glycogen synthesis; reduce glycogen decomposition; promote protein synthesis; reduce protein decompositon and fat accumulation; increase beneficial bacteria and reduce harmful bacteria in the intestine; increase glucose utilization in fat and muscle groups. In addition, *Lactobacillus* increases the number of short-chain fatty acids (SCFAs) producing bacteria, enhances bile acid synthesis and Cholesterol reverse transport, promotes the production and absorption of vitamins, thereby showing therapeutic activity against sarcopenia. M1, M1 macrophages; M2, M2 macrophages; Tregs, Regulatory T cells; Th17, T helper cell 17 (Created with figdraw.com).

For example, it has been observed that the conditioned medium (CM) from *L. rhamnosus* JY02 reduced myotube atrophy caused by dexamethasone (DEX) and lowered the expression of muscle degradation markers, MuRF1 and atrogin-1, in C2C12 cells. Meanwhile, *L. rhamnosus* JY02 supplement could enhance the expression of muscle-enhancing markers such as MHC Iβ, MHC IIα, and Myo-D, decreases the levels of muscle degradation markers, and mitigates muscle atrophy symptoms in a murine model (Lee et al., 2023). Moreover, *Lactobacillus casei* LC122 increased the mass of quadriceps femoris (QM) and gastrocnemius (GM) muscles, and enhanced muscle strength and function in aged mice (Ni et al., 2019). These results sparked tremendous interest, suggesting that priority consideration may be involved in opting for *Lactobacillus* probiotics as an adjunct treatment for sarcopenia, and motivated us to explore the underlying mechanisms. The relief of the inflammatory state, the clearance of excess reactive oxygen species, the improvement of skeletal muscle metabolism, the regulation of gut microbiota and its metabolites has been identified as a potential mechanism through which *Lactobacillus* can alleviate sarcopenia (Chen L. et al., 2022; He et al., 2023; Hor et al., 2021; Yan et al., 2019) (Figure 1). These findings can not only provide new ideas for the treatment of sarcopenia, but also further deepen our understanding of the role of *Lactobacillus* in the gut-muscle axis.

### 3.1 The abundance and diversity of *Lactobacillus* in gut microbiota

*Lactobacillus*, a gram of gram-positive anaerobic bacteria recognized for its capacity to ferment glucose and other sugars to generate lactic acid. Classified within the Firmicutes phylum, it falls under the Bacillus class, *Lactobacillales* order, and *Lactobacillus* family. It encompasses several subspecies including *Lactobacillus* fermentum (*L. fermentum*), *Lactobacillus plantarum* (*L. plantarum*), *Lactobacillus salivarius* (*L. salivarius*), *Lactobacillus acidophilus* (*L. acidophilus*), *Lactobacillus rhamnosus* (*L. rhamnosus*), etc (Heeney et al., 2018). It is estimated that this genus is believed to represent roughly 0.3% and 6% among the total bacterial population within the human colon and duodenum, respectively (Almonacid et al., 2019; Nistal et al., 2016). Among the over 200 known lactobacilli species, only a few have been consistently and frequently interacted with the human gastrointestinal tract. However, the number of discovered species has significantly increased recently, with more than 50 lactobacilli species now being repeatedly verified in the feces of healthy volunteers (Rossi et al., 2016). Among them, the most abundant lactobacilli species primarily include *L. casei*, *Lactobacillus delbrueckii* (*L. delbrueckii*), *L. rhamnosus*, *L. plantarum*, *Lactobacillus mushroomorum* (*L. mushroomorum*).

It has been demonstrated that a positive correlation existed between the levels of gut *Lactobacillus* and skeletal muscle mass index. In the elderly, it has been shown that the proportion of certain gut microbiota in patients with sarcopenia has changed, with a significant decrease in the proportion of Lactobacilli, Bacteroidetes, and Prevotella, along with an increases in *Escherichia coli* (Lou et al., 2024). In the gut of accelerated aging mice, the enriched bacterial genera, including Odoribacter, Oscillibacter, and Anaerotruncus, were found to be negatively correlated with muscle and mitochondrial function. At the same time, these bacteria were positively correlated with pro-inflammatory cytokines and negatively correlated with anti-inflammatory cytokines (Chen L. et al., 2022). *Lactobacillus* can restore the composition and beta diversity of intestinal microbiota, which may be one of the ways to play a therapeutic role in sarcopenia (Picca et al., 2019). For example, *L. paracasei*, can increase the populations of Akermaceae and Deinococcaceae, which are associated with muscle weight and Myosin Heavy Chain (MyHC) expression. In contrast, it can reduce the populations of Odobacteraceae and certain members of Deinococcaceae, which are associated with elevated levels of Interleukin-6 (IL-6) and Muscle RING-Finger 1 (MuRF1) expression (Baek et al., 2023). In addition to increasing beneficial gut bacteria, *Lactobacillus* can enhance epithelial integrity through adequate colonization, making it less susceptible to infections by harmful bacteria such as *Bacteroides*, *Escherichia*, and *Shigella*, as well as their translocation into the intestinal lumen (Karczewski et al., 2010). It also has the ability to provide nutritional support (SCFAs, vitamins, amino acids, and amines, etc.) and lower the pH value of the intestinal cavity, thereby preventing the colonization of harmful bacteria and maintaining the gut microbiota balance (Hong et al., 2018). Consequently, the role of *Lactobacillus* in preserving balance in gut environments should not be underestimated.

### 3.2 Regulation of immunity and inflammation

When the gut microbiota balance of sarcopenia patients was in a state of disorder, their gut immune microenvironment was also disrupted. As the primary defense against microbiota, the intestinal mucosa contains a variety of immune cells, which work together to preserve immune homeostasis in both the peripheral blood and the intestinal mucosal environment. Imbalance in the gut microbiota can lead to abnormal activation of the immune system and a state of chronic inflammation (Zheng et al., 2020). The process of muscle wasting during aging can be exacerbated by abnormalities in the immune system, while the chronic low-grade inflammation caused by immune is considered one of the pivotal factor contributing to sarcopenia (Saini et al., 2016). *Lactobacillus* may modulate excessive activation of intestinal and systemic inflammatory responses from various angles by altering the number, recruitment, and differentiation of immune cells, thereby limiting the progression and worsening of sarcopenia.

*Lactobacillus* may alleviate symptoms of muscle atrophy by restoring the quantity and proportion of altered immune cells. Firstly, *Lactobacillus reuteri* enhanced anti-inflammatory force, hindered pro-inflammatory power, and inhibited muscle atrophy by restoring impaired systemic immunosuppressive function through upregulating the Treg/Th17 ratio. The transcription factor Foxp3 regulates Treg development, while the activity of Tregs is modulated by IL-35 and IL-10. Respectively, *L. reuteri* not only restored the levels of T lymphocytes (CD3γ), Th1 lymphocytes (Tbet), and Th17 lymphocytes (IL-17A) but also maintain levels of Foxp3 and IL-10 to raise the frequency of Tregs in tissue (Bindels et al., 2016). Besides *L. reuteri*, *L. rhamnosus* was also effective in impacting the relative abundance of immune cells due to it could repair and maintain intestinal barrier function by upregulating the levels of TJs and GLP-2, ultimately regulating Th17/Treg and related cytokine levels in mesenteric lymph nodes (Guo et al., 2023).

In addition to changing the quantity and proportions of various immune cells, the immune cell phenotype was also induced changes to regulate the inflammatory by *Lactobacillus*. The changes in macrophage immune metabolism are related to muscle wasting symptoms. In the context of skeletal muscle functional metabolic disorders, the phenotype of macrophages changes from M2 (anti-inflammatory) to M1 (pro-inflammatory) (Liu N. et al., 2023). M1 and M2 represent two phenotypes in macrophages with completely opposite functions, The former is characterized by high expression of pro-inflammatory cytokines, including IL-1β, IL-6, IL-12, and TNF-α, while the latter predominantly produces anti-inflammatory cytokines like IL-10 and TGF-β (Shapouri-Moghaddam et al., 2018). *L. rhamnosus* GroEL modulates macrophage phenotype to relieve the inflammatory pressure in muscle by inhibiting markers associated with M1-like macrophages and upregulating M2-like macrophage markers (Dias et al., 2021). Apart from the change in macrophage markers, *L. rhamnosus* GG can also significantly decrease the mRNA expression of MCP-1 and CD11c, associated with monocyte recruitment and M1 macrophage activation, thereby suppressing the infiltration and activation of M1 cells (Park et al., 2015).

From a dialectical perspective, a correlation existed between the distribution of immune cells and the production of inflammatory cytokines. With numerous immune cells releasing inflammatory cytokines, these cytokines may in turn hinder their recruitment and function. Many experiments have demonstrated that *Lactobacillus* can alter inflammatory cytokine levels. *L. casei* Shirota (LcS), boosted the levels of IL-10 and short-chain fatty acids (SCFAs), suppressed macrophage production of TNF-α and lowered TNF-α levels (Chen L. et al., 2022). Moreover, other *Lactobacillus* strains exhibit similar effects. *L. reuteri*, *L. acidophilus*, *L. fermentum*, *L. brevis* and *L. rhamnosus* enhanced IL-10 levels while decreasing TNF-α expression (Archer et al., 2021; Peña and Versalovic, 2003). Meanwhile, *Lactobacillus* upregulated the expression of anti-inflammatory mediators. A multifunctional factor involved in immune modulation, G-CSF, which exhibited a negative correlation with inflammation-promoting mediators like TNF-α, IL-12 and IL-23, which were proven to have potential in skeletal muscle repair and regeneration (Wright et al., 2017). *L. reuteri* and *L. gasseri* escalated the expression of G-CSF and reduced markers of atrophy including Atrogin-1, MuRF1, LC3, and Cathepsin L. Increasing IL-10 decreased the generation of pro-inflammatory factors like IFN-γ and IL-2/IL-1β, which alleviated the inflammatory response and inhibiting the progression of sarcopenia (Bindels et al., 2012).

### 3.3 Relief of oxidative stress

Oxidative stress is closely related to cell damage and aging (Marzetti et al., 2013). Reactive oxygen species (ROS), primarily composed of superoxide anions, hydrogen peroxide, and hydroxyl radicals, are crucial components of the neutrophils antibacterial library. Excessive ROS can lead to oxidative stress in muscle tissue with activating intracellular signaling pathways involved in sarcopenia (Bonetto et al., 2009). Oxidative stress can induce lipid and protein oxidation, disrupt the composition and structural integrity of muscle cell membranes, thereby leading to a decline in skeletal muscle function (König et al., 2001). Thus, managing oxidative stress could be an effective approach to treating sarcopenia.

Various studies have verified the high antioxidant capacity of *Lactobacillus* strains. The antioxidant properties of *Lactobacillus* are closely associated with its ability to scavenge reactive oxygen species and increase antioxidant levels (Lin and Chang, 2000). Firstly, *L. paracasei* can promote the expression of antioxidant-associated genes superoxide dismutase (SOD) and catalase (CAT), inhibits the oxidation process, and prevents inflammation (Lin et al., 2021). *L. fermentum* not only upregulates the expression of antioxidant genes, but also eliminates peroxides in tissues through glutathione peroxidase (GPx) activity to alleviate oxidative stress that damages muscles and protect muscle (Hor et al., 2021). Secondly, *L. plantarum* has the ability to modulate the nuclear factor erythroid 2-related factor 2 (Nrf2) signaling pathway, thereby enhancing the body’s antioxidant capacity and reducing the levels of malondialdehyde (MDA), a biomarker of oxidative stress (Chen Q. et al., 2022). Thirdly, mitochondria serve as key sites for ROS production. Dysfunctional mitochondria increases inflammation and ROS that are strongly linked to sarcopenia (Picca et al., 2018), which may also be a new target for *Lactobacillus* therapy. *L. plantarum* can regulate mitochondrial biogenesis, including PGC1 α, SIRT1, NRF1, and TFAM prevent mitochondrial dysfunction to reduce inflammation and ROS production, ultimately mitigating sarcopenia in the aging mouse model (Chen et al., 2019). Additionally, *Lactobacillus* strains play a significant role in combating muscle aging by modulating AMPK activity and mitochondrial regulation related genes. As a key regulator of cellular energy balance, AMPK not only modulates mitochondrial dynamics but also helps counteract oxidative stress by reducing energy expenditure and conserving resources, thereby promoting the restoration of muscle function. Through this pathway, they influence the expression of the p53 gene, which encodes a tumor suppressor protein. P53 is an important marker for senescence caused by telomere stress and regulates critical processes such as cell cycle arrest, DNA repair, apoptosis, and cellular aging (Qian and Chen, 2013). Specifically, these strains (such as LP-0291 and LF-DR9) can reduce the expression of the p53 gene in local gastrocnemius and tibialis muscles, which is associated with the activation of AMPK. This series of mechanisms provides effective support in resisting aging, highlighting the significant potential of *Lactobacillus* strains in relief of oxidative stress (Hor et al., 2019).

Additionally, excessive NO can break the balance of intracellular redox and trigger inflammatory reactions, resulting in muscle tissue damage and atrophy (Kaminski and Andrade, 2001). NO is a key player in muscle tissue inflammation, injury, and atrophy, as verified in animal research (Hall et al., 2011). *Lactobacillus* alleviates oxidative stress by indirectly or directly inhibiting the production of NO. *L. plantarum* down-regulates the expression level of the syncytin-1, nitric oxide synthase (iNOS) and TNF-α gene present in skeletal muscle, restores overall energy balance in muscle tissue of animals and reduces the oxidative response (Yi et al., 2021). Beside, arginine deaminase, an enzyme found in *L. brevis*, aids in the inhibition of NO is produced by competing with NOS for arginine, its common substrate, and metabolizing it into citrulline and ammonia (Zhang et al., 2014). Moreover, *Lactobacillus* can not only restore normal NO and GSH levels, but also stimulate the proliferation of myogenic stellate cells; thus having an anti-aging-related myopathy effect (Abdel-Halim et al., 2023).

### 3.4 Regulation of the skeletal muscle metabolism

#### 3.4.1 Glucose

Skeletal muscle is a vital organ for glucose metabolism, and the composition of the gut microbiome influences glycogen storage in muscles. Microbiome dysregulation can reduce muscle glucose utilization, leading to insufficient glycogen reserves, which in turn affects muscle function and quality, accelerating muscle atrophy (Daily and Park, 2022). *Lactobacillus* may directly or indirectly affect glucose metabolism, thereby improving muscle function. In the liver, GSK-3 β is a serine/threonine kinase that can inhibit glycogen synthase (GS) activity, and *L. acidophilus* can downregulate the expression of GSK-3 β and increase glycogen synthesis (Yan et al., 2019). As the main metabolites of *Lactobacillus* and other gut microbiota, SCFAs have been proven to promote the synthesis of muscle glycogen (Fushimi and Sato, 2005). Regarding glycogen consumption, fermented milk enriched with probiotics that contains *L. acidophilus* and *L. casei* could reduce fasting blood glucose (FBG) and HbA1c levels, stimulate muscle glucose absorption (Hu et al., 2017).

Sarcopenia is often accompanied by insulin resistance and both are mutually pathogenic, resulting in a vicious cycle (Liu and Zhu, 2023). *Lactobacillus* strains contribute to a decrease in insulin resistance, consequently againsting the progression of sarcopenia. Glucose transporter 4 (GLUT4) in skeletal muscle can mediate insulin-stimulated glucose uptake (Herman and Kahn, 2006). In mice with diet-induced obesity, the administration of *Lactobacillus* lowered insulin resistance and improved glucose tolerance, potentially by alleviating endoplasmic reticulum stress in skeletal muscle, inhibiting macrophage activation, and enhancing GLUT4 expression (Tabuchi et al., 2003). Furthermore, a mixture of probiotic content *L. rhamnosus*, *L. acidophilus* and Bifidobacterium bifidum regulated insulin signal transduction in muscles, improving insulin sensitivity and glucose tolerance in obese mice. This strategy fully restored Akt phosphorylation levels in muscle tissue while significantly reducing the relative amounts of TNF-α and IL-6 transcripts (Bagarolli et al., 2017).

#### 3.4.2 Protein

Skeletal muscle serve as a storage site for amino acids stored in the form of proteins (Baskin et al., 2015). The balance between the synthesis of skeletal muscle proteins (MPS) and the breakdown of muscle proteins (MPB) is crucial for skeletal muscle phenotype, mass and function (Schiaffino et al., 2013). Increasing protein degradation and reducing protein synthesis is the main mechanism of muscle atrophy in sarcopenia (Sartori et al., 2021). Dysbiosis in the gut microbiota can lead to increased gut barrier permeability, endotoxin translocation, and insulin resistance, resulting in impaired muscle protein synthesis. Supplementing with *L. plantarum* can regulate gut microbiota dysbiosis, reduce the expression of muscle atrophy markers such as Atrogin-1 and LC3 protein in mice, and promote muscle protein synthesis (Chen et al., 2016). *L. plantarum* and *L. rhamnosus* not just modulate the structure of gut microbiota and its metabolites, but regulating the expression of protein synthesis genes mTOR and S6K in skeletal muscle, ultimately enhancing nitrogen metabolism in the weaned piglets (He et al., 2023). In addition, whey protein fermented with *L. gasseri* prevented dexamethasone (DEX)-induced muscle atrophy by stimulating myogenesis and protein synthesis through activation of the IGF-1-PI3K/AKT/mTOR pathway, reducing protein breakdown via FOXO-mediated modulation of the ubiquitin-proteasome pathway (UPP) and autophagy lysosomal pathway (ALP). Specifically, it prevented the increase in mRNA and protein expression of in UPP related molecules atrogin-1/MAFbx and MuRF1, as well as those of ALP related molecules LC3, calpain L, and BNI (Jang et al., 2023). *L. paracasei* also exert beneficial effects on skeletal muscle by modulating key signaling pathways involved in muscle protein homeostasis. Specifically, these probiotics inhibit FOXO3a activation, which in turn suppresses the expression of MuRF1 and MAFbx/Atrogin-1, two E3 ubiquitin ligases that regulate ubiquitin-mediated skeletal muscle protein degradation. Additionally, probiotics inhibit NF-κB activation, a pathway known to promote muscle atrophy (Zhong et al., 2023). There by enhancing AKT and mTOR activation, pathways that play a central role in promoting myogenic gene expression and protein synthesis. This dual action reducing protein degradation and enhancing protein synthesis leads to an increase in MyHC (myosin heavy chain) expression, a key marker of muscle differentiation and contractile function. Together, these mechanisms contribute to the preservation of muscle mass, strength, and function (He et al., 2023; Zhang et al., 2024). Collectively, these findings indicate that *Lactobacillus* show the potential to prevent muscle atrophy and promote overall muscle protein homeostasis.

#### 3.4.3 Lipid

Clinically, sarcopenia is often accompanied by obesity, which can further promote the occurrence of sarcopenia (Tardif et al., 2014). This type of obese muscle atrophy includes intramuscular fat accumulation and muscle fibrosis. Abnormal accumulation of lipids in the body will accelerate muscle atrophy and muscle steatosis (Vial et al., 2020). Skeletal muscle lipid metabolism is also regulated by lactobacilli. Animal experiments have found that the increase in skeletal muscle fat content in rats fed with a high-fat high-sucrose (HFS) diet may be related to a decrease in the relative abundance of *Lactobacillus* and Prevotella (Collins et al., 2016). *L. plantarum* can improve lipid oxidation by increasing the mRNA expression of lipid oxidation genes, including CPT1, ACOX1, and MCAD, and reduce lipid synthesis by activating the SIRT1-PGC-1 alpha pathway, leading to reduction of fat accumulation (Kwon et al., 2020). Additionally, supplementing with *L. plantarum* can reduce white adipose tissue without weight gain, enhance muscle mass, and increase the number of type I fibers related to exercise endurance in the gastrocnemius muscle, thereby preventing muscle atrophy caused by muscle function degradation (Chen et al., 2016).

Adiponectin (APN) is the most abundantly expressed human body adipokine, having a role in resisting inflammation and regulating muscles (Krause et al., 2019). A meta-analysis on the association between adiponectin level and sarcopenia has shown that individuals with sarcopenia had lower adiponectin levels compared to those without sarcopenia (Komici et al., 2021). APN may activate signals by binding to T-cadherin, thereby promoting muscle regeneration and resisting muscle atrophy (Tanaka et al., 2019). *L. rhamnosus* effectively increases insulin sensitivity and reduces lipid accumulation by stimulating APN secretion and AMPK activation, while upregulating expression genes involved in fatty acid oxidation, such as PPAR-a, CPT1, and ACOX in the liver and skeletal muscle of HFD mice (Kim et al., 2013). Interestingly, measurements of total body fat, fat deposition, and lipid levels in diet-induced obese mice showed no difference between the control group and mice supplemented with *Lactobacillus acidophilus* (Arora et al., 2012). Therefore, the efficacy of a *Lactobacillus* is highly strain-specific. Diverse strains of lactobacilli have different effects and ways on lipid metabolism, which may explain differences in the results of studies using various strains.

### 3.5 Influence on the metabolites of gut microbiota

#### 3.5.1 Short-chain fatty acids (SCFAs)

**FIGURE 2:**
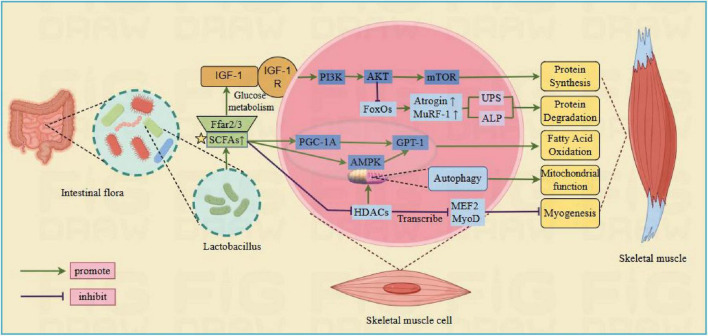
*Lactobacillus* regulate skeletal muscle pathways through short-chain fatty acids. Ffar2/3, free fatty acid receptors 2 and 3; IGF-1, insulin like growth factor; IGF1R, IGF-1 membrane receptor; MTOR, rapamycin target protein; CPT-1, carnitine palmitoyltransferase 1; PGC-1α, peroxisome proliferator-activated receptor gamma coactivator 1-alpha; PI3K, phosphatidylinositol kinase; AMPK, adenylate activated protein kinase; FoxO, forked transcription factor; MuRF-1, muscle specific ring finger protein 1; Atrogin, human muscle atrophy protein Fbox; HDAC, histone deacetylase; MEF2, myocyte enhancer factor 2; MyoD, myogenic differentiation antigen (Created with figdraw.com).

SCFAs is primarily consisting of acetic, propionic, and butyric acids, which are produced by specific anaerobic colon bacteria to decompose carbohydrates. These SCFAs are vital in supporting gut homeostasis, glucose, protein and lipid metabolism, immune system, and body inflammatory response (Tan et al., 2014). Current studies propose that SCFAs can participate in muscle regulation through the following pathways (Figure 2): (1) SCFAs are able to attach to free fatty acid receptors 2 and 3 (FFAR2/3), enhancing glucose metabolism and IGF-1 release. IGF-1 activates the PI3K/Akt/mTOR signaling cascade, supporting muscle protein synthesis and phosphorylates FoxO to prevent muscle protein breakdown (Pekmez et al., 2019). (2) SCFAs increase mitochondrial adenylate activating proteins. AMP-activated protein kinase (AMPK) enhances the activity of carnitine palmitoyltransferase 1 (CPT-1), leading to increased fatty acid oxidation. (3) Regulation is carried out via peroxisome proliferator-activated receptor gamma coactivator 1-alpha (PGC-1α), which functions as an activator for numerous nuclear receptors and transcription factors, regulating the expression of CPT-1 to increase fatty acid oxidation (Zong et al., 2002). In mouse models, the positive effects of CPT-1 and PGC-1α on muscle mass have been confirmed, showing their ability to inhibit muscle atrophy (Hénique et al., 2015). (4) SCFAs passively diffuse into cells to inhibit HDACs, which possess key roles in the function and metabolism of skeletal muscle, affecting the development and differentiation of muscle cells by regulating the acetylation status of myogenic regulatory factors such as MyoD, MEF2 and Myogenin. This involves the promotion of skeletal muscle atrophy, inhibition of mitochondrial synthesis, and regulation of glucose and lipid oxidation processes (Tian et al., 2020).

Beneficial gut bacteria, including *Lactobacillus*, Bifidobacteria, Firmicutes, Bacteroidetes, Actinobacteria and Enterococcus faecalis, promote the production of SCFAs, which regulate skeletal muscle metabolism and muscle fiber phenotype through the gut-muscle axis, indirectly influencing muscle health (Anachad et al., 2023). *Lactobacillus* are key growth factors for gut bacteria that produce short chain fatty acids. They not only produce acetic acid on its own, but also regulate the bacteria that produce SCFA in the intestine, promoting the mutual transformation of SCFA through cross fertilization. For example, bacteria that produce butyric acid can increase SCFA levels by utilizing lactic acid and acetic acid (Morrison et al., 2006). The concentrations of acetate, propionate, and butyrate in the cecal contents of mice significantly increased after oral supplementation of *L. plantarum*. In acetate-producing bacteria, there was a significant increase in the abundance of members from the Peptococcaceae family and the Ruminococcaceae genus UCG-004. Furthermore, there is a significant rise in the abundance of bacteria producing butyrate, particularly members of the Lachnospiraceae family. This leads to improve SCFAs levels and relieve sarcopenia. Meanwhile, the presence of *L. plantarum* increased the abundance of beneficial SCFA producing bacteria and significantly reduced the number of intestinal pathogenic bacteria (such as Proteobacteria and Enterobacteriaceae) (Lee C.-C. et al., 2021).

The vast majority of ongoing research aims to quantify the levels of SCFA influenced by LAB. Nevertheless, its effect on muscle is complex. In addition to concentration, further research is needed into the individuals and the ratio. Different SCFAs mediated by *Lactobacillus* have different effects on muscle fibers. Propionic acid shows a positive association with the ratio of type I muscle fibers, but the cross-sectional area of type I muscle fibers is negatively correlated with the ratio of type IIb muscle fibers. The ratio of butyric acid to IIa type muscle fibers is positively correlated (Liu T. et al., 2023). In the intestine, the molar ratio of acetic, propionic, and butyric acids is roughly 60:20:20, respectively (Markowiak-Kopeć and Śliżewska, 2020). *In vitro* studies have shown that SCFAs exhibit a dose-dependent effect, and glucose uptake in C2C12 myotubes varies in different proportions of combinations. Single SCFA does not increase glucose uptake (Otten et al., 2023). Moreover, although lactic acid may not fall under the category of SCFAs, it is an essential compound generated by *Lactobacillus* and a critical precursor for the production of SCFAs. The link between lactic acid and muscle atrophy needs to be further explored.

#### 3.5.2 Lipopolysaccharide (LPS)

A healthy intestinal barrier function can reduce inflammation and promote nutrient absorption, thereby helping to prevent the occurrence of sarcopenia (Baumgart and Dignass, 2002). The gut mechanical barrier, as the first line of defense against the penetration of harmful substances and pathogens (such as lipopolysaccharide) into the body, is primarily consisted of small cells that form a protective layer over the intestinal epithelium, interconnected by adherens junctions (AJs) and tight junctions (TJs). This intricate network serves as the principal determinant of paracellular permeability within the gastrointestinal tract (Drolia and Bhunia, 2019). Tight junctions (TJs) contain transmembrane proteins, including occludin and claudins, as well as peripheral membrane proteins like ZO (occlusive zone protein) as important complexes for sealing the space between Intestinal Epithelial Cells (IEC), maintaining cell polarity, and sustaining the osmotic function of the intestinal barrier. Dysfunction of the intestinal barrier can cause bacteria and their metabolites (such as LPS) in the intestine to leak out and enter the bloodstream.

LPS, including lipid A, core oligosaccharides, and O antigen, is a strong endotoxin present in the outer membrane of Gram negative bacteria. When excessive LPS is transferred to the systemic circulation and increases plasma LPS levels, it can trigger inflammation, thereby facilitating the onset and progression of sarcopenia (Bindels and Delzenne, 2013). Due to the expression of Toll-like receptors (TLRs) by skeletal muscle cells, they can identify a variety of pathogen-associated molecular patterns (Boyd et al., 2006). Nuclear factor κB (NF-κB), the transcription factor involved in muscle-specific activation, induces sarcopenia, thereby triggering the toll-like receptors/NF-κB pathway (Thoma and Lightfoot, 2018). Therefore, upon recognition of LPS from gut bacteria by TLR4 on skeletal muscle cells, nuclear factor-kappa B (NF-κB) is activated, leading to the production of interleukin-6 (IL-6) and tumor necrosis factor-alpha (TNF-α) (Barton and Medzhitov, 2003). The IL-6 secreted by macrophages and T-cells is involved in the inhibition of protein synthesis (Haddad et al., 2005), and the TNF-a producted by macrophages induces protein degradation and apoptosis in cells during the initial stages of stress response (Li et al., 2005). Another pathway shown that LPS promotes the expression of Atrogin-1 and MuRF1 genes via MAPK activation, activating both the UPS and autophagy-lysosome pathways, leading to skeletal muscle protein degradation (Ronnebaum et al., 2014).

*Lactobacillus* hold a pivotal role in repairing and preserving the intestinal barrier function, maintaining intestinal homeostasis, which affect the occurrence and progression of muscle atrophy. It can protect the gut barrier by stimulating the proliferation of intestinal epithelial cells and the expression of TJ protein related growth factors, and balance inflammatory factors through changing downstream signaling pathways after specific recognition of receptors. TJs were observed downregulated in early-weaned piglets infected with *Escherichia coli* k88, leading to increased permeability of the intestinal mucosa. However, the administration of *L. casei* salvaged this disaster. *L. casei* showed a remarkable ability to reinstate the expression of TJs, such as ZO-1, occludin, and claudin-1 in piglets, and enhanced the intestinal structure. Furthermore, *L. casei* can also promote the regeneration and repair of intestinal mucosal epithelial cells by enhancing their proliferation, thus protecting the integrity of intestinal epithelial tissue. Simultaneously it increased the thickness of intestinal muscles and the height of villi, further improving the overall health of the intestine (Wang et al., 2019). As a probiotic mixture consisting of Plant *Lactobacillus* and Fermented *Lactobacillus*, which has been proven to have the ability to increase TJs expression and reduce the levels of LPS in their plasma in high-fat diet-fed mice. It also can enhance GLP-1 levels, alleviate endoplasmic reticulum (ER) stress, improve peripheral insulin sensitivity, and increase skeletal muscle mass (Balakumar et al., 2018).

In terms of inhibiting LPS induced inflammatory factors, *L. rhamnosus* (LGG) exhibited anti-inflammatory effects in the gastrointestinal tract. Certain *Lactobacillus* species demonstrated a specific ability to mitigate LPS-induced inflammatory damage in mouse models (Petrof et al., 2009). In human smooth muscle cell (SMC), the protective effect of LGG against LPS-induced damage is mediated by inhibiting phosphorylation in both NF-κB subunits and suppressing the secretion of pro-inflammatory cytokine IL-6 upon activation of surface TLR2, whose expression is reduced following exposure to LGG. Furthermore, LGG also reinstates the secretion levels of the anti-inflammatory cytokine IL-10 (Ammoscato et al., 2013).

#### 3.5.3 Bile acids (BAs)

BAs, including chenodeoxycholic acid (CDCA) and cholic acid (CA), are endogenously synthesized in the liver from cholesterol. After undergoing lipid digestion and absorption in the duodenum, the lipids journey to the distal ileum and the proximal colon. Here, they undergo a transformation into free bile acids, facilitated by the action of gut bacteria and bile salt hydrolase (BSH). A small portion of BA that has not entered the enterohepatic circulation is metabolized by gut microorganisms in the colon, producing non-conjugated bile acids and secondary bile acids through isomerization and dehydroxylation (Cai et al., 2022). Previous studies have shown that the level of 12α-hydroxylated cholic acid (such as deoxycholic acid) is negatively correlated with skeletal muscle volume. In contrast, the level of non-12α-hydroxylated cholic acid (such as chenodeoxycholic acid) is positively correlated with skeletal muscle volume (Kobayashi et al., 2017). It has been also revealed that low-dose ursodeoxycholic acid (UDCA) blocks the production of cancer induced reactive oxygen species through the PKC signaling pathway, thereby inhibiting transcription factors AP-1 and NF-κ B, leading to the expression of pro-inflammatory cytokine TNF-α and reducing the consumption of cachexia (e.g., fat and skeletal muscle tissue loss) (Tschirner et al., 2012).

**FIGURE 3:**
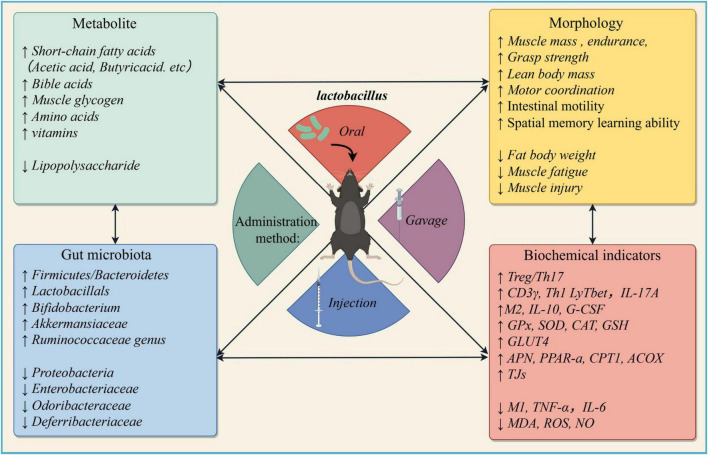
Clinical findings of muscle atrophy mice/rat models after *Lactobacillus* intervention (Created with figdraw.com)).

*Lactobacillus* can modulate BAs metabolism and affect its gastrointestinal transport. LAB possess bile acids hydrolase, which exhibits tolerance and deconjugation activity toward BAs, allowing them to survive and exert bile acids dissociation ability (Hernández-Gómez et al., 2021). Joyce et al. (2014) demonstrated the importance of gut microbiota in bile acid metabolism and systemic gene expression. Mice treated with *Lactobacillus plantarum* exhibited enhanced expression levels of genes related to bile acid synthesis and reverse cholesterol transport in the liver. Additionally, the expression of the bile acid receptor TRG5 was increased in the liver, ileum, and skeletal muscle of mice treated with *L. plantarum* (Kwon et al., 2020). The binding of bile acid with TGR5 can enhance D2 activity (Watanabe et al., 2006), resulting in elevated T3 levels in skeletal muscle cells, thereby facilitating the development and regeneration of skeletal muscle (Mullur et al., 2014). Whereas, given the significant differences in bile acid profiles between mice and humans, further studies will be necessary to determine the correlation between bile acids, gut microbiota and muscle atrophy in humans. Accordingly, exploring the underlying mechanisms still remains many challenges (Figure 3).

#### 3.5.4 Vitamins (Vits)

A balanced gut microbiota such as *Lactobacillus* and Bifidobacterium can delivers essential vitamins, including B-vitamins B3, B5, B6, B7, B12, folate, and vitamin K. There is an inextricable link between various vitamins and sarcopenia, with adequate vitamin intake being crucial for maintaining muscle mass and function, The production of these compounds by gut microbiota can enter the systemic circulation and ultimately affect skeletal muscle cells, particularly in the case of vitamin D (Bauer et al., 2013; Uchitomi et al., 2020). Low plasma vitamin B12 and vitamin D has been observed in elderly patients with sarcopenia (Ates Bulut et al., 2017). *Lactobacillus* can influence the production, absorption, and metabolism of vitamins, thereby preventing sarcopenia. It can regulate skeletal muscle synthesis and metabolism by restoring a normal gut microbiome, producing folate and vitamin B12. This process helps prevent oxidative stress and endothelial damage caused by hyperhomocysteinemia, thereby preventing skeletal muscle dysfunction (Langille et al., 2014). Similarly, *Lactobacillus* strains can also influence vitamin D levels. Study found that *L. reuteri* NCIMB 30242 may promote vitamin D absorption by increasing liver 25-hydroxylase activity or 7-dehydrocholesterol (7-DHC) concentration, thereby leading to an increase in the production of 25(OH)D (Jones et al., 2013). Research has also found that *L. rhamnosus* GG and *L. plantarum* are capable of increasing the expression of the vitamin D receptor (VDR) protein in both mouse and human intestinal epithelial cells while enhancing its transcriptional activity. By activating the VDR signaling pathway, these probiotics ultimately promote vitamin D absorption and the expression of its target genes, such as the antimicrobial peptide cathelicidin, thereby exerting anti-inflammatory effects (Wu et al., 2015). In another study, it was also found that *L. rhamnosus* GG can promote gut absorption of cholecalciferol, and increase 25-Hydroxyvitamin D3 levels by upregulating the protein levels of vitamin D transporters in senile osteoporosis, thereby affecting the absorption of vitamin D and treating osteoporosis (Cheng et al., 2022). Many studies have demonstrated a highly significant link between sarcopenia and osteoporosis, with both conditions share similar pathological mechanisms (Wang et al., 2024). This gave rise to the term “osteosarcopenia.” Although current research on *Lactobacillus* is either focused on osteoporosis (Bose and Sharan, 2024; Guo et al., 2023; Huang et al., 2022) or sarcopenia (Kim et al., 2023; Lee et al., 2023; Ni et al., 2019; Rondanelli et al., 2022). Considering the strong association and shared pathophysiology between osteoporosis and sarcopenia, *Lactobacillus*, due to its ability to enhance vitamin D production, absorption, promote vitamin D metabolism, and improve calcium utilization, presents great potential in the treatment of osteosarcopenia.

## 4 *Lactobacillus*-related clinical prevention and treatment in sarcopenia

The gut microbiota is essential for maintaining overall human health and preventing diseases. Table 1 lists more detail on the actual effectiveness of each strain. In recent years, direct supplementation of *Lactobacillus* has gradually become a suitable treatment option to prevent or alleviate sarcopenia and improve patient quality of life, which is considered as an effective means of assisting or replacing traditional drug therapy. Several randomized controlled trials have substantiated the efficacy of various probiotics, with a particular emphasis on *Lactobacillus*. These studies have been conducted across diverse populations, focusing notably on adult and elderly individuals suffering from sarcopenia. In terms of promoting muscle health, *L. plantarum* PL-02 and LY-66 has demonstrated benefits in increasing muscle mass and reducing body fat accumulation, which can help mitigate myosteatosis, muscle steatosis is a key factor contributing to sarcopenia, thereby effectively preventing its progression (Lee et al., 2024). Long-term and excessive exercise can lead to microdamage of muscle fibers and inflammatory responses. Patients may develop muscle dysfunction/insufficiency, and a small proportion may even suffer from muscle atrophy (Rubio-Arias et al., 2019). Supplementing *Lactobacillus* can effectively alleviate these adverse conditions. Two capsules (3 × 10^10^ CFU/capsule) of *L. plantarum* PS128 administered each morning and evening before meals for four weeks reduced muscle damage (i.e., myoglobin, LDH, and CPK), significantly elevated the antioxidation indicator SOD, and reduced muscle inflammation response of marathon runners (Fu et al., 2021). The same dose of *L. plantarum* TWK10 administered over 6 weeks reduced muscle fatigue and improved endurance. In terms of body composition, the administration of TWK10 resulted in favorable changes in body composition (body fat, bodyweight, muscle weight, BMI). Particularly in the high-dose group, a significant decrease in body fat and increases in muscle mass was observed in healthy participants without professional athletic training (Huang et al., 2019).

**TABLE 1:** Effect of *Lactobacillus* on organism.

Strain	Experiment type	Dose and methods	Application time	Outcome	References
*L. rhamnosus* JY02	*In vivo* (mice)	1 × 108 CFU/mouse/d by oral	5 weeks	Decreased IL-8, TNF-α. increased IL-10, MHC Iβ, MHC Iiα, Myo-D	Lee et al., 2023
	*In vitro* (cell)	0.01%–10%JY02-CM	1 weeks	Inhibited expression of MuRF1 and atrogin-1	Lee et al., 2023
*L. rhamnosus* ATCC7469	*In vivo* (rat)	1 × 109 CFU/mL/d	6 weeks	Decreased Th17/Treg ratio, TNF-α and IL-17; increased IL-10, TGF-β, TJs and GLP-2	Guo et al., 2023
*L. rhamnosus* ATCC 53103	*In vitro* (cell)	Log 10 6 CFU/mL	24 h	Inhibiting NF-κ phosphorylation of B subunit and activation of surface TLR2 promote the secretion of pro-inflammatory cytokine IL6	Ammoscato et al., 2013
*L. rhamnosus* GG	*In vivo* (mice)	1 × 108 CFU/mouse/d	4 weeks	Decreased MCP-1 and CD11c	Park et al., 2015
	*In vivo* (rat)	2% lyophilized GG cells	9 weeks	Reduced insulin resistance and boosted glucose tolerance by reducing endoplasmic reticulum stress in skeletal muscle	Tabuchi et al., 2003
	*In vivo* (mice)	1 × 108 CFU/mouse/d	13 weeks	Upregulated expression genes, such as PPAR-a, CPT1, and ACOX	Kim et al., 2013
*L. rhamnosus* BSL or R23	*In vivo* (rat)	1 × 109 CFU/mL/d	4 weeks	Inhibited expression of glucose-6-phosphatase	Farida et al., 2020
*L. casei* LC122	*In vivo* (mice)	2 × 109 CFU/mouse/d	12 weeks	Increased claudin 1, ZO-1, JamA and Defa; decrease Sik1 and Pgc1a4.	Ni et al., 2019
*L. casei* Shirota	*In vivo* (mice)	1 × 108 or 1 × 109 CFU/mouse/d	12 weeks	Increased IL-10 and short-chain fatty acids (SCFAs), inhibited macrophage TNF-α	Chen L. et al., 2022
*L. casei* Zhang	*In vivo* (rat)	4.0 × 109 CFU/rat/d	2 weeks	Inhibited NO production	Zhang et al., 2014
	*In vivo* (piglet)	1 × 107 CFU/g/d diet	2 weeks	Repair of intestinal mucosal epithelial cells	Wang et al., 2019
*L. casei* DK211	*In vivo* (human)	37 g/2/d	8 weeks	Increased branched chain amino acids and improved muscle protein synthesis	Kim et al., 2023
*L. reuteri* 100-23	*In vivo* (mice)	2 × 108 CFU/ml	2 weeks	Restored CD11c, CD3γ, Tbet and IL-17A; decreased IFN-γ, TNF-α and IL-1β	Bindels et al., 2016
*L. reuteri* GroEL	*In vivo* (mice)	1 ng per 10 μl by intrarectal injection	4 days	Inhibited pro-inflammatory M1-like macrophages markers, and favored M2-like markers	Dias et al., 2021
*L. fermentum* MCC2759	*In vivo* (rat)	1 × 109 CFU/ml	8 weeks	Improved gut barrier integrity (ZO-1) and insulin sensitivity	Archer et al., 2021
*L. fermentum* DR9	*In vivo* (mice)	1 × 10^10^ CFU/d	12 weeks	increased GPx activity and upregulated the expression of SOD and CAT	Hor et al., 2021
*L. mixture* GG, MTCC 5690/5689	*In vivo* (mice)	1.5 × 109 colonies/mouse/d	16 weeks	Reduce LPS levels, increase GLP-1 levels, alleviate ER stress, increase insulin sensitivity and skeletal muscle mass	Balakumar et al., 2018
*L. gasseri* 311476	*In vivo* (mice)	2 × 108 CFU/ml, oral	13 days	Reduced Atrogin-1, MuRF1, LC3 and Cathepsin L; increased IL-10, IL-4 and G-CSF	Bindels et al., 2012
*L. gasseri* IM13	*In vitro* (cell)	10, 25, and 50 μg/mL	48 h	Stimulated myogenesis and protein synthesis, reduced protein breakdown	Jang et al., 2023
*L. paracasei* GKS6	*In vivo* (mice)	5.0 × 109 CFU/kg BW/d	14 weeks	Increased muscle grip strength and mass, upregulate antioxidant genes	Lin et al., 2021
*L. paracasei* PS23	*In vivo* (mice)	1 × 109 CFU/mouse/d	12 weeks	Prevents mitochondrial dysfunction and ROS production	Chen et al., 2019
*L. paracasei* P62	*In vivo* (mice)	1 × 109 CFU/mouse/d	8 weeks	Increased grip strength and treadmill running distance and time, also increased AKT activation, PGC1α, SIRT1 and MyHC expression	Baek et al., 2023
*L. plantarum* KSFY01	*In vivo* (mice)	1.0 × 109 CFU/kg	10 weeks	Increased the activity of antioxidant enzymes, decreased the level of MDA	Chen Q. et al., 2022
*L. plantarum* CQPC02	*In vivo* (mice)	1.0 × 108 and 1.0 × 109 CFU/kg, gavage	4 weeks	Down-regulated the expression of the syncytin-1, iNOS and TNF-α	Yi et al., 2021
*L. plantarum* TWK10	*In vivo* (mice)	2.05 × 108 and 1.03 × 109 CFU/kg, oral	6 weeks	Reduce the expression of Atrogin-1 and LC3 protein and promote muscle protein synthesis	Chen et al., 2016
	*In vivo* (mice)	1 × 109 CFU/mouse/d	8 weeks	Increased muscle strength, muscle glycogen and scfas levels; Regulate gut microbiota	Lee C.-C. et al., 2021
	*In vivo* (human)	2 × 10^10^ or 6 × 10^10^ CFU/day	18 weeks	increased muscle mass, left hand grip strength, lower limb muscle strength, and improved gait speed and balance	Lee M.-C. et al., 2021
	*In vivo* (human)	3 × 10^10^ CFU/capsule	6 weeks	Reduced muscle fatigue and body fat; improved endurance; increased muscle mass	Huang et al., 2019
*L. plantarum* PS128	*In vivo* (human)	3 × 10^10^ CFU/capsule	4 weeks	Improved myoglobin, LDH, and CPK; elevated SOD; reduced muscle inflammation response	Fu et al., 2021
*L. plantarum* HY7715	*In vivo* (mice)	1 × 108 CFU/kg/day	5 weeks	Restores the gut microbiome composition and beta diversity shift	Lee K. et al., 2021
*L. plantarum* JL01	*In vivo* (piglet)	1 × 109 CFU/mL	28 days	Regulated the expression of protein synthesis gene and the structurt of gut microbiota	He et al., 2023
*L. acidophilus*	*In vivo* (rat)	1 × 109 CFU/ml/d	4 weeks	Stimulated the proliferation of myogenic stellate cells and reduced oxidative stress	Abdel-Halim et al., 2023
	*In vivo* (mice)	1.8 × 109 CFU	5 weeks	Restored Akt phosphorylation levels in muscles; Decreased TNF-α and IL-6	Bagarolli et al., 2017
*L. acidophilus* KLDS1.1003	*In vivo* (mice)	1 × 109 CFU/d	6 weeks	Reduced glycogen synthase kinase and oxidative stress; increased insulin sensitivity	Yan et al., 2019
*L. plantarum* Q180	*In vivo* (mice)	1 × 109 or 1 × 10^10^ CFU/mL	12 weeks	Increases the expression of genes involved in bile acid synthesis and cholesterol reverse transport; upregulated the expressions of adiponectin in adipose tissue, irisin in skeletal muscle, SAT and FGF21	Kwon et al., 2020
*L. fermentum* DR9 and *L. sakei* Probio 65	*In vivo* (rat)	1 × 10^10^ CFU/mL	12 weeks	Reduced p53 gene expression in localized gastrocnemius muscle and tibia.	Hor et al., 2019

With aging, muscle mass and function gradually decline in the elderly population, making them more susceptible to sarcopenia. In this context, *Lactobacillus* can play a significant role by reducing muscle fat infiltration, improving metabolic processes, and exerting anti-inflammatory effects, thereby effectively slowing down the progression of sarcopenia. In obese or metabolic syndrome patients, where muscle fat infiltration is more prevalent due to altered lipid metabolism, *Lactobacillus* can help regulate body composition and improve lipid metabolism. Additionally, for individuals engaged in intense physical training, such as athletes or fitness enthusiasts, supplementation with *Lactobacillus* offers benefits in alleviating exercise-related muscle damage while maintaining overall muscle health. This is particularly important for those who are consistently subjected to high-intensity workouts that may contribute to muscle strain and inflammation. These effects not only mitigate the risk of sarcopenia but also support overall metabolic health in these populations.

The ability of *Lactobacillus* to improve muscle function and quality can serve as a preventive measure. Similarly, these capabilities, such as enhancing muscle protein synthesis and reducing inflammation, also play a therapeutic role in alleviating sarcopenia symptoms in elderly or chronic disease patients. Twice daily administration of whey protein fermented by *L. casei* DK211 in capsule ingested (37 g per group) an 8-week period of consumption treatment increased levels of branched chain amino acids in plasma, and improved muscle protein synthesis, especially muscle strength and exercise performance in 48 middle-aged healthy males relative to the placebo-treated control group (Kim et al., 2023). In the same vein, another study with a similar *L. casei* treatment protocol on elderly subjects with sarcopenia found analogous changes, while reduced inflammation in the muscle tissue and restored muscle function after administration of a new food composed of omega-3 fatty acids, leucine and probiotic *L. paracasei* PS23 (Rondanelli et al., 2022). In addition, it was found that supplementing TWK10 for 6 weeks possess a trend of enhancing muscle mass, grip strength, lower limb muscle strength, gait speed and balance in Frail Older Adults. The effect became even more pronounced as the supplementation duration extends to the 18th week (Lee M.-C. et al., 2021). The above studies that demonstrate how *Lactobacillus* supplementation can both prevent muscle wasting in at-risk populations and aid in recovery in individuals already experiencing sarcopenia.

## 5 Limitation and prospect

While *Lactobacillus* strains show promise in mitigating sarcopenia, current research still has notable limitations that need to be addressed. *Lactobacillus*-mediated microbiota and metabolites perform an essential foundational role. Currently, the majority of studies still rely on 16S sequencing technology to determine the classification information of microbial communities, which may reduce the precision of specific categories and ignore the role of fungus and viruses from the gut microbiota. Such an integrated approach could reveal additional factors that contribute to sarcopenia, paving the way for novel therapeutic interventions that target a broader spectrum of the microbiome. Therefore, subsequent research should incorporate fungi and viruses in the intestines to provide a thorough analysis of key microorganisms that might interact with *Lactobacillus* to mitigate sarcopenia. For instance, shotgun metagenomic sequencing technology that can investigate species level and microbial community functional changes at a more detailed classification level (Ma et al., 2020). Furthermore, the connection between specific bacterial communities and substance metabolism is not yet fully understood, with most research primarily identifying correlations. In the future, *in vitro* fermentation simulations, biosensor technology should be applied to detect the generation of metabolites and their influence on sarcopenia.

Although *Lactobacillus* strains are generally recognized as safe and widely used, mild gastrointestinal symptoms such as bloating and gas are occasionally reported. In certain cases, especially in individuals with compromised immune systems, *Lactobacillus* may overgrow and cause bacterial translocation. This involves the migration of bacteria from the gut to other parts of the body, such as the bloodstream or internal organs, potentially leading to infections and even rare cases of systemic infections including bacteremia, have occurred (Doron and Snydman, 2015; Sanders et al., 2016). Give to this, it is need to develop personalized probiotic formulations, tailored to an individual’s health status, microbiome composition, and specific needs, can optimize benefits and minimize adverse effects by ensuring that the selected *Lactobacillus* strains are compatible with each individual’s gut microbiome. Due to different strains of *Lactobacillus* exhibit varying safety profiles and functional properties such as *Lactobacillus hilgardii* X1B, which produces putrescine and agmatine through arginine and ornithine, may be harmful to the human body, especially at high concentrations, which may cause hypertension, allergic reactions, or combine with nitrite to form carcinogenic nitrosamines (Arena and Manca de Nadra, 2001). Therefore, careful strain selection is crucial. For example, strains with a long history of safe use, such as *L. rhamnosus* GG or *L. plantarum* may be preferred for certain populations. Though different strains share many similarities, the unique characteristics of different subspecies result in variations in the anti-sarcopenic properties and functional components of each stain (Baek et al., 2023). Thus, combining the utilization of different strains of *Lactobacillus* to develop more efficient probiotics or to jointly use *Lactobacillus* with other probiotics or prebiotics, it may be possible to reduce the required dosage of *Lactobacillus*, thereby minimizing the risk of adverse effects. In recent years, newly emerged microorganisms, including Akkermansia muciniphila, Ruminococcus gnavus, and others, may be regarded as powerful candidates to deliver a partnership with *Lactobacillus*. However, the effectiveness of *Lactobacillus* in clinical applications is still hindered by issues such as low vitality and low bioavailability in the gastrointestinal process. As we focus on the combination, an effective delivery method seems to double our efforts with half the effort. Emerging nanotechnology can design various forms of nanoparticles (NPs) have been designed for probiotics/prebiotics/symbiotics or their different combinations, facilitating the efficient coupling of probiotics with intestinal mucosal tissues and ensuring their orderly targeted release (Dangi et al., 2023). Moreover, a new packaging method for probiotics method [encapsulate a single bacterium with Gum arabic (GA) and Maltodextrin] tremendously increase the survivability of *Lactobacillus acidophilus* under the gastrointestinal conditions (Arepally et al., 2020).

## 6 Conclusion

We summarized the potential mechanisms of *Lactobacillus* in treating sarcopenia, which demonstrated that *Lactobacillus* play both direct and indirect roles in alleviating the symptoms of sarcopenia. Specifically, *Lactobacillus* play a pivotal role in regulating immunity, inhibiting inflammation and oxidative stress, hosting metabolism, balancing skeletal muscle metabolism and adjusting gut microecology, all of which interact with each other and protect muscle mass and function in sarcopenia. Although there are medications available for treating other clinical conditions that can be used for sarcopenia, their safety and efficacy remains limited, especially in patients with severe sarcopenia. In contrast, *Lactobacillus* offers a more effective treatment with fewer side effects and lower cost burden. This review demonstrated that *Lactobacillus* has the potential to become a promising option for the treatment of sarcopenia.
